# Valgus Slipped Capital Femoral Epiphysis in Patient with Hypopituitarism

**DOI:** 10.1155/2017/8981250

**Published:** 2017-01-05

**Authors:** Yoshihiro Kotoura, Yasuhiro Fujiwara, Tatsuro Hayashida, Koji Murakami, Satoshi Makio, Yuichi Shimizu, Yoshinobu Oka, Wook-Choel Kim, Taku Ogura, Toshikazu Kubo

**Affiliations:** ^1^Department of Orthopaedic Surgery, Nantan General Hospital, Nantan, Japan; ^2^Department of Orthopaedics, Graduate School of Medical Science, Kyoto Prefectural University of Medicine, Kyoto, Japan

## Abstract

Slipped capital femoral epiphysis (SCFE) is a common disease of adolescent and the epiphysis is positioned more posteromedially in relation to the femoral neck shaft with varus SCFE; however, posterolateral displacement of the capital epiphysis, valgus SCFE, occurs less frequently. We report a case of valgus SCFE in a 17-year-old boy with hypopituitarism. After falling down, he experienced difficulty in walking. The radiographs were inconclusive; however three-dimensional computed tomography images showed lateral displacement of the epiphysis on the right femoral head. Valgus SCFE was diagnosed. The patient underwent in situ pinning of both sides. In situ pinning on the left side was performed as a prophylactic pinning because of endocrine abnormalities. At the 1-year follow-up, he could walk without any difficulty and there were no signs of pain. The epiphysis is commonly positioned more posteromedially in relation to the femoral neck shaft with most SCFE, but, in this case, the epiphysis slipped laterally. Differential diagnosis included femoral neck fracture (Delbet-Colonna type 1); however, this was less likely due to the absence of other clinical signs. Therefore, we diagnosed the patient as SCFE. When children complain of leg pain and limp, valgus SCFE that may not be visualized on anteroposterior radiographs needs to be considered.

## 1. Introduction

Acute posttraumatic limp is a common reason for children's hospitalization; however the diagnosis is not always easy for trauma physicians, because sometimes the signs are not specific and its diagnostic images are unclear. Many of these causes are not dangerous, but sometimes there are conditions requiring more serious treatment. One of these conditions is slipped capital femoral epiphysis (SCFE) and the treatment is emergent. SCFE is a common disease of adolescent and the epiphysis is usually positioned more posteromedially in relation to the femoral neck shaft with varus SCFE. Varus SCFE is not uncommon; however posterolateral displacement of the capital epiphysis, valgus SCFE, is uncommon. A definitive diagnosis can be difficult but there should be a high degree of clinical suspicion. We report a case of valgus SCFE in a 17-year-old boy with hypopituitarism. 

## 2. Case Report

The patient was a 17-year-old boy with hypopituitarism, autism, and a history of septooptic dysplasia with whom communication was difficult. He was receiving treatment with dexamethasone 100 *μ*g and Levothyroxine sodium 1 mg per day. One month before admission, he experienced a fall, which resulted in difficulty in walking. At the first visit to the physician, because he could not describe any symptoms, only knee radiography was performed and there were no abnormalities. The pain in his right leg progressively worsened and he presented to our outpatient clinic after one month.

**Figure 1:**
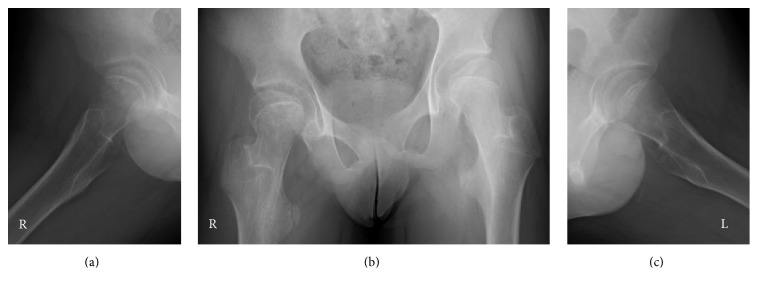
(a) Frog-lateral radiograph of the right hip joint showing almost normal. (b) Anteroposterior radiograph of the right hip joint showing lateral displacement of the right epiphysis and valgus neck shaft angles bilaterally. (c) Frog-lateral radiograph of left hip showing normal.

**Figure 2:**
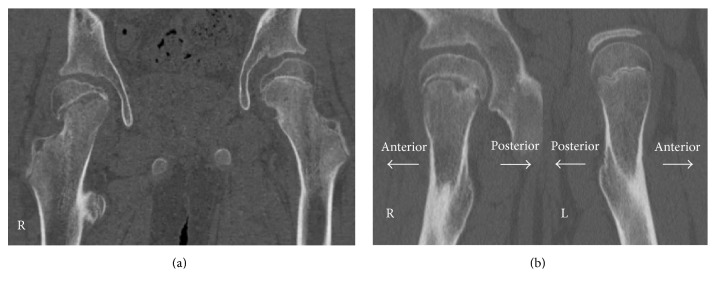
Reconstruction coronal (a) and sagittal (b) CT images of the right hip showing lateral displacement of the epiphysis.

**Figure 3:**
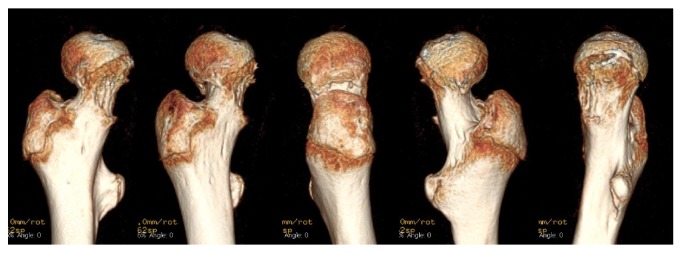
3D CT images showed the right epiphyseal displacement was laterally.

On physical examination, the patient was slightly obese with a height of 161 cm and weight of 63 kg noted. His right leg was externally rotated, and he hesitated to stand up. The passive range of motion of the right hip was limited, especially in adduction and internal rotation (right/left; flexion 90°/120°, extension 10°/10°, abduction 45°/45°, adduction 0°/30°, external rotation 60°/45°, and internal rotation 10°/30°). Serum insulin-like growth factor-1 and insulin-like growth factor-binding protein 3 levels were low, but free triiodothyronine, free thyroxine, and adrenocorticotropic hormone levels were almost normal. The radiographs of the hip joints showed a right atypical slippage of the epiphysis (Figure 1). Both neck shaft angles were large (right/left; 160°/155°) and lateral tilts of the physis were normal on intact side (right/left; 35° lateral/13° medial). Three-dimensional computed tomography (3D CT) images showed lateral epiphyseal displacement on the right femoral head (Figures 2 and 3), indicating valgus SCFE.

**Figure 4:**
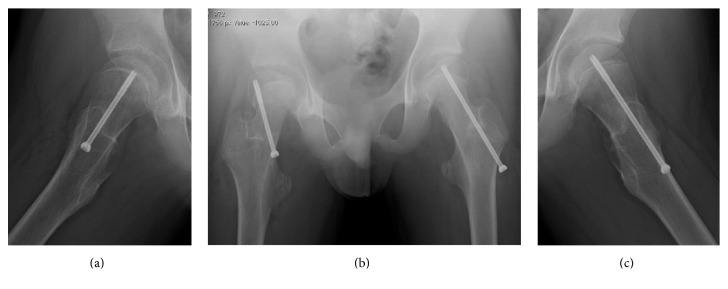
Postoperative (a) frog-lateral radiograph of the right hip joint. (b) Anteroposterior radiograph showing the screw for the valgus SCFE was placed more medially than for a typical varus SCFE. (c) Frog-lateral radiograph of the left hip joint.

The patient underwent in situ pinning of the hip on both sides. On the right side, the procedure was performed through a limited 5 cm longitudinal incision, positioned medially on the thigh (frog position), posterior to the femoral neurovascular bundle. A single *φ*5.5 mm partially threaded cannulated screw (Meira, Japan) was placed across the physis. In situ pinning on the left side was performed as a prophylactic pinning because of endocrine abnormalities (Figure 4). Postoperatively, we had difficulty in limiting weight bearing due to communication difficulties.

One month after surgery, he could walk better than before the operation. At the 1-year follow-up, he could walk without difficulty and there were no signs of pain. The passive range of motion of the right hip joint was slightly improved in flexion, adduction, and internal rotation (100°, 20°, and 20°, resp.). Radiographs showed no signs of avascular necrosis or chondrolysis of the epiphysis; the physis showed no signs of early partial closure.

## 3. Discussion

SCFE is an adolescent hip disorder. Clinically, the patient with SCFE may have hip pain, thigh pain or knee pain, an acute or insidious onset of a limp, and decrease of the range of motion of the hip. The condition is associated with obesity and growth surge and it is occasionally associated with endocrine disorders such as hypothyroidism, growth hormone deficiency, hypopituitarism, and hyperparathyroidism. In most SCFE cases, the capital femoral epiphysis is displaced posterior and medial relative to the femoral neck. The medial displacement shows a varus appearance on anteroposterior radiographs. Lateral displacement of the femoral head is a rare case and is referred to as “valgus” SCFE. Müller first described valgus SCFE in 1926 [1]. Fewer than 100 cases have been reported to date, and these case reports have not described the specific criteria [2] used in the diagnosis of valgus SCFE. Loder et al. estimated the prevalence of valgus slip to be 1.9% by taking into account all the reported cases in literature until 2006 [3]. This indicated approximately 1-2% of all idiopathic SCFE cases were probably due to a valgus slip. Yngve et al. reported that the valgus neck shaft angles and lateral tilt of the physis were risk factors for valgus SCFE [4]. According to the report by Loder et al., there were significant differences between valgus and varus SCFE for symptom duration, body mass index, and sex: 76% of valgus SCFE occurred in girls [5].

The delayed skeletal maturation is a common finding in endocrine disorders and the risk factor for SCFE. According to the report by Loder et al., 5.2 to 6.9% of patients with SCFE are associated with endocrine disorders [6]. They assessed 85 patients with SCFE and known endocrine disorders. The most common primary endocrine diagnosis was hypothyroidism in 34 patients (40%) and growth hormone deficiency in 21 patients (25%) and 30 patients (35%) had other endocrinopathies such as hypogonadism, hypopituitarism, hyperparathyroidism, and growth hormone excess. Yngve et al. reported on the association between endocrine disorders and valgus SCFE, 2 patients with underdeveloped genitalia and 1 with panhypopituitarism in a series of 7 patients with valgus patients [4]. In a review by Shank et al., four of the 12 valgus patients suffered from panhypopituitarism and they indicated the association between hypopituitarism and valgus SCFE [2]. In this case, the valgus neck shaft angles and hypopituitarism resulting in growth hormone deficiency and hypothyroidism were consistent with those previously reported.

The Klein line has been reported to be an early indicator of varus SCFE. Klein et al. used the line along the superior aspect of the femoral neck as an index of SCFE [7]. In valgus SCFE, however, the line will always be normal and this has emphasized the need for lateral radiographs to be performed in all children with hip pain [3]. In our case, the Klein line was normal on anteroposterior radiograph. In addition, the lateral view was almost normal on lateral radiograph. 3D CT images were helpful and clearly showed that the direction of the epiphyseal displacement was laterally. Venkatadass et al. reported that 3D CT images showed slippage direction of the femoral head and needed for appropriate treatment selection [8]. Differential diagnoses included femoral neck fracture (Delbet-Colonna type 1 [9]) and coxofemoral dysplasia; however these were less likely because of the patient's age and absence of other clinical signs.

Segal et al. have described the importance of appropriate screw placement when considering treatment options [10]. In a valgus slip, the slippage is further posterior and hence the entry point for the screw must be more medial [8]. Both Segal and Loder reported that the proximity of the neurovascular bundle increases the risk associated with the medial approach and recommended an open surgery to protect the neurovascular structures. In this case, a mini-incision, which was positioned medially on the thigh (frog position), was performed. We also used in situ pinning through the medial femoral metaphysis. During surgery, a three-dimensional imaging system (ARCADIS Orbic 3D®, Siemens, Munich, Germany) was used to visualize the direction of slippage and to guide the appropriate placement of screws.

In conclusion, acute posttraumatic limp and leg pain in the adolescent boy was diagnosed as valgus SCFE. It might be associated with valgus neck shaft angles and endocrine disorders. 3D CT images were useful in establishing diagnosis. For appropriate and safe in situ pinning, we made a limited open incision and used three-dimensional imaging system to protect the femoral neurovascular bundle. When children complain of leg pain, valgus SCFE which may not be visualized on anteroposterior radiographs needs to be considered.
